# Brewing an Interaction: Non-commercial Green Tea-Associated Supratherapeutic Tacrolimus Levels Presenting With Gastrointestinal Symptoms

**DOI:** 10.7759/cureus.109952

**Published:** 2026-05-30

**Authors:** Rangesh Modi, Freny Patel, Aenasha Chag, Megha R Joshi

**Affiliations:** 1 Gastroenterology and Hepatology, The University of Chicago Medicine, Chicago, USA; 2 Internal Medicine, Pramukhswami Medical College, Karamsad, Karamsad, IND; 3 Internal Medicine, Smt. Nathiba Hargovandas Lakhmichand Municipal Medical College, Ahmedabad, IND; 4 Internal Medicine, Woodhull Medical and Mental Health Center, New York, USA

**Keywords:** crohns colitis, green tea extract (gte), post liver transplant, tacrolimus toxicity, upper gastrointestinal symptoms

## Abstract

Tacrolimus is a calcineurin inhibitor with a narrow therapeutic index and can have significant interactions with foods and herbs that affect CYP3A4 and P-gp metabolism. We report a case of gastrointestinal symptoms associated with supratherapeutic tacrolimus levels due to heavy consumption of non-commercial green tea with orange extract in a 44-year-old woman with Primary Sclerosing Cholangitis status post liver transplant and refractory Crohn's disease. She presented with worsening abdominal pain, nausea, and bloating while on a stable tacrolimus dose with previously therapeutic trough levels. Evaluation showed a supratherapeutic tacrolimus trough of 18.3 ng/mL without evidence of infection, hepatic dysfunction, or acute inflammatory bowel disease flare. Endoscopic findings were unchanged from prior examinations. Tacrolimus was held and green tea discontinued, leading to rapid normalization of tacrolimus levels and complete symptom resolution within 24 hours without corticosteroid escalation. Tacrolimus was later resumed at the prior dose with stable therapeutic levels during follow-up. This case highlights the potential for non-commercial herbal preparations, including green tea products, to alter tacrolimus metabolism and cause clinically significant toxicity.

## Introduction

Tacrolimus (FK506) is a macrolide lactone immunosuppressant that inhibits calcineurin, thereby suppressing T-cell activation and cytokine transcription [1]. Given its narrow therapeutic index and substantial interpatient pharmacokinetic variability, therapeutic drug monitoring is essential. Tacrolimus exposure is influenced by CYP3A4/CYP3A5 metabolism, P-glycoprotein (P-gp) transport, hepatic function, systemic inflammation, and interactions with drugs, foods, and herbal supplements [1]. Tacrolimus is widely used in solid organ transplantation and immune-mediated gastrointestinal diseases such as inflammatory bowel disease (IBD). Its gastrointestinal toxicity is nonspecific and may mimic underlying disease activity, complicating recognition in patients with complex intestinal disorders.

Herbal supplement use is increasingly common among patients receiving immunosuppressive therapy. Non-commercial preparations are of particular concern due to variability in composition, including bioactive compounds and potential additives. Several dietary substances, including grapefruit, pomelo, and pomegranate, can alter tacrolimus metabolism via CYP3A and P-gp inhibition [2]. Green tea contains catechins and theaflavins that may also modulate these pathways, although clinical evidence remains limited and inconsistent [2]. Reported interactions with green tea are rare, and the effects of concomitant orange extract or other additives in non-commercial preparations remain unknown.

We report a case of supratherapeutic tacrolimus levels and gastrointestinal toxicity associated with a non-commercial green tea preparation containing orange extract in a patient with Crohn’s disease and previously stable tacrolimus trough concentrations.

## Case presentation

A 44-year-old White woman with primary sclerosing cholangitis post living donor liver transplant (age 38), and Crohn’s colitis post total colectomy with ileorectal anastomosis with a residual short sigmoid colon segment (age 34) presented for one month of gradually worsening abdominal pain, nausea, and bloating. She had six non-bloody liquid bowel movements per day, which was her baseline post-colectomy. She denied vomiting, blood in stools, weight loss, or fevers. Her Crohn’s colitis was diagnosed at age 17, and she needed multiple different biologic classes and agents through the years but had persistent inflammation due to primary or secondary loss of response to biologics.

She was currently on subcutaneous vedolizumab 108 mg every two weeks and budesonide 6 mg daily for her Crohn’s, and her last flexible sigmoidoscopy (six months prior) showed a normal neo-terminal ileum but an inflamed anastomosis and rectum with biopsies showing moderate active Crohn’s in the rectosigmoid colon. Her anti-rejection medications included mycophenolate 500 mg twice a day and tacrolimus immediate release (IR) 2 mg twice a day, with the last tacrolimus levels being 4.5 ng/mL (goal <6 ng/mL) a month ago. Her tacrolimus dose had been stable for the past six months. She denied any other over-the-counter medication or supplement use. She mentioned consuming over five to six cups/day of non-commercial green tea with orange extract made by a local herbalist for the past three weeks to help with inflammation and stopped consuming green tea one day prior to admission.

On admission, she was afebrile, HR was 85/minute, BP was 127/89 mm Hg, and RR was 16/minute. Physical examination was unremarkable except for a soft, mildly distended abdomen with normal bowel sounds. Initial complete blood count (CBC) and comprehensive metabolic panel (CMP) were unremarkable except for mild leukocytosis of 13.4 x10^3^/µL (normal range, 3.5-11). Serum C-reactive protein (CRP) was normal. Her initial tacrolimus trough level was 18.3 ng/mL (goal<6). Table 1 summarizes laboratory findings.

**Table 1:** Summary of laboratory results, with abnormal values shown in bold. U: units; L: liter; µL: microliter; dL: deciliter; mg: milligram; mmol: millimole; mEq: milliequivalent; mL: milliliter; g: gram; ng: nanogram

Laboratory type	Test (blood)	Measured value	Reference range in SI units
Complete blood count	White blood cells	13.4 x10^3^/µL	3.5-11 x10^3^/µL
	Hemoglobin	12.8 g/dL	13.5-17.5 g/dL
	Platelet	270 x10^3^/µL	150-450 x10^3^/µL
Renal function	Blood urea nitrogen	11 mg/dL	7-20 mg/dL
	Creatinine	0.87 mg/dL	0.5-1.4 mg/dL
Metabolic profile	Glucose	99 mg/dL	60-99 mg/dL
	Sodium	141 mmol/L	135-145 mmol/L
	Potassium	4.1 mEq/L	3.5-5 mEq/L
	Chloride	108 mmol/L	98-108 mmol/L
	Bicarbonate	24 mmol/L	23-30 mmol/L
	Calcium	9 mg/dL	8.4-10.2 mg/dL
	Magnesium	2.1 mg/dL	1.6-2.5 mg/dL
	Phosphate	3.2 mg/dL	2.5-4.4 mg/dL
Liver function	Aspartate aminotransferase	20 U/L	8-35 U/L
	Alanine aminotransferase	32 U/L	8-35 U/L
	Alkaline phosphatase	82 U/L	50-150 U/L
	Total bilirubin	0.3 mg/dL	0.1-1 mg/dL
	Total protein	7 g/dL	6-8.3 g/dL
	Albumin	4.1 g/dL	3.5-5 g/dL
Others	C-reactive protein	3 mg/L	<5 mg/L
Tacrolimus trough (goal <6)	Day 1	18.3 ng/mL	5-15 ng/mL
	Day 2	8.7 ng/mL	
	Day 3	4.8 ng/mL	

**Figure 1:**
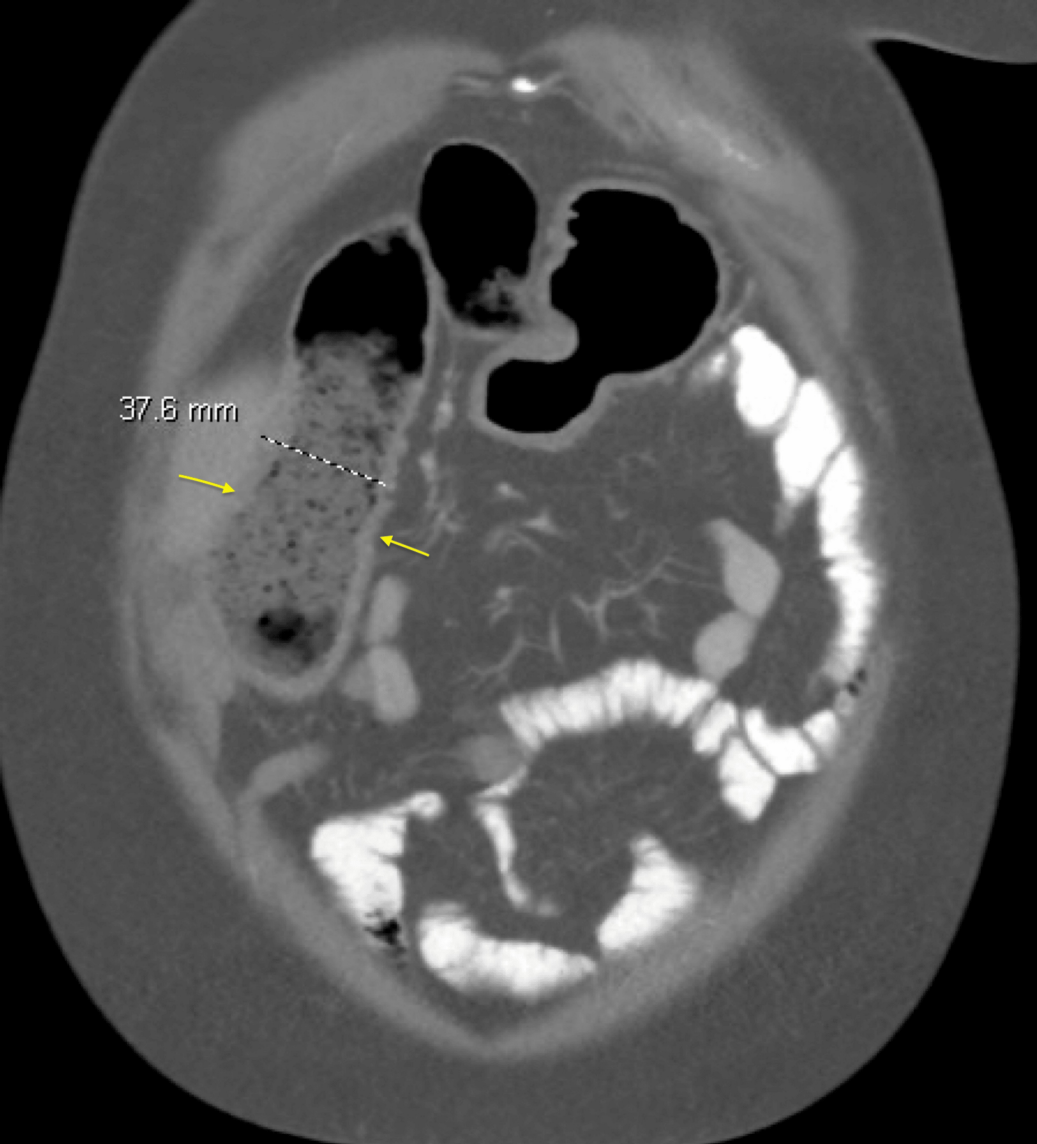
Computed tomography (CT) scan of the abdomen and pelvis with intravenous (IV) contrast, coronal section, demonstrating a distended small-bowel loop measuring 3.8 cm in diameter (yellow arrows).

Stool gastrointestinal pathogen PCR and *Clostridium difficile* toxin test were negative. A CT scan of the abdomen and pelvis with IV contrast showed feces-filled, mildly distended (3.8 cm), and thick-walled distal small bowel loops (coronal section as shown in Figure 1), indicating fecal stasis and post-surgical changes of liver transplant and total colectomy with ileorectal anastomosis.

**Figure 2:**
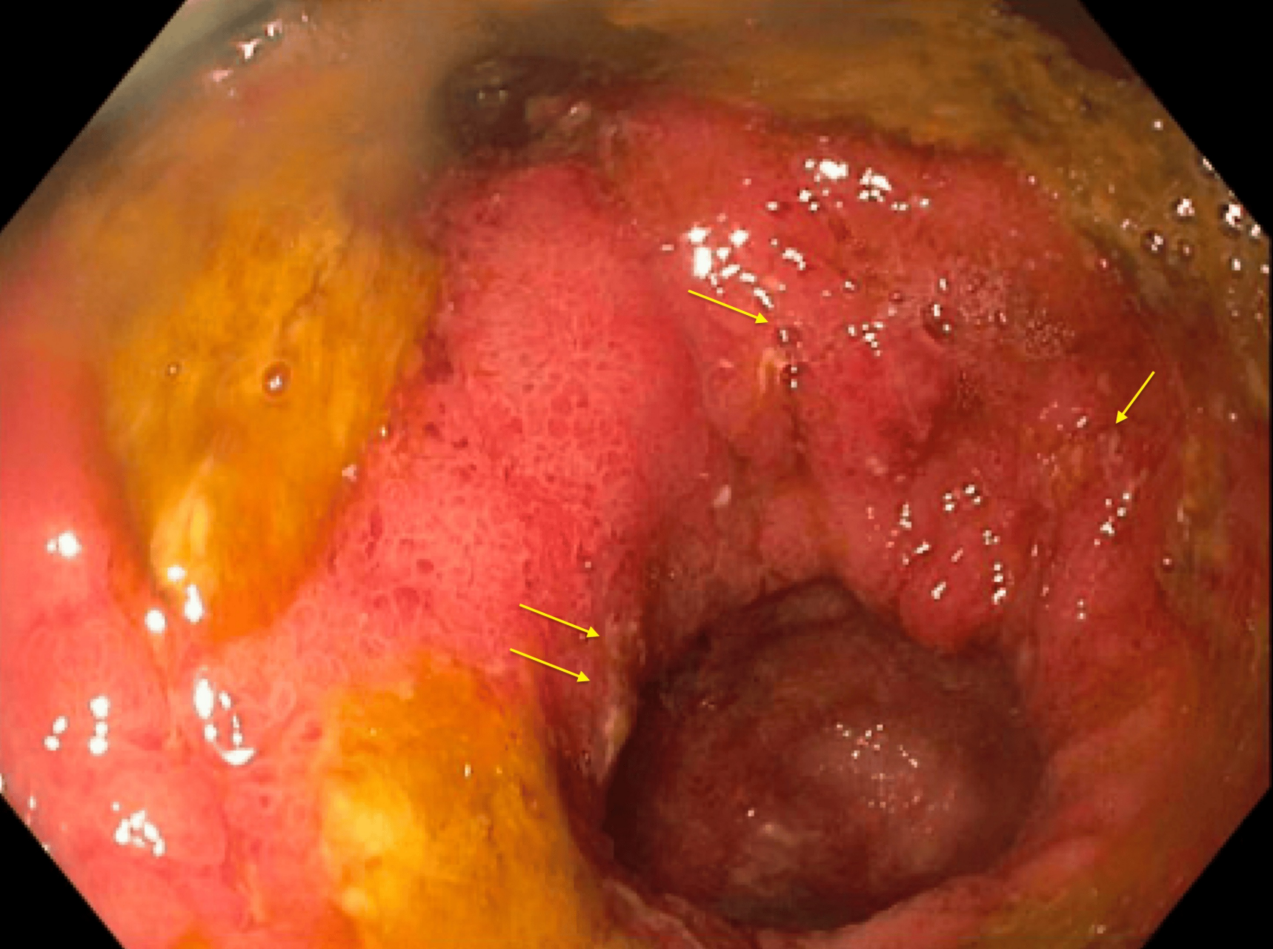
Ileorectal anastomosis demonstrating granular, congested mucosa with inflammatory at the anastomosis (yellow arrows).

**Figure 3:**
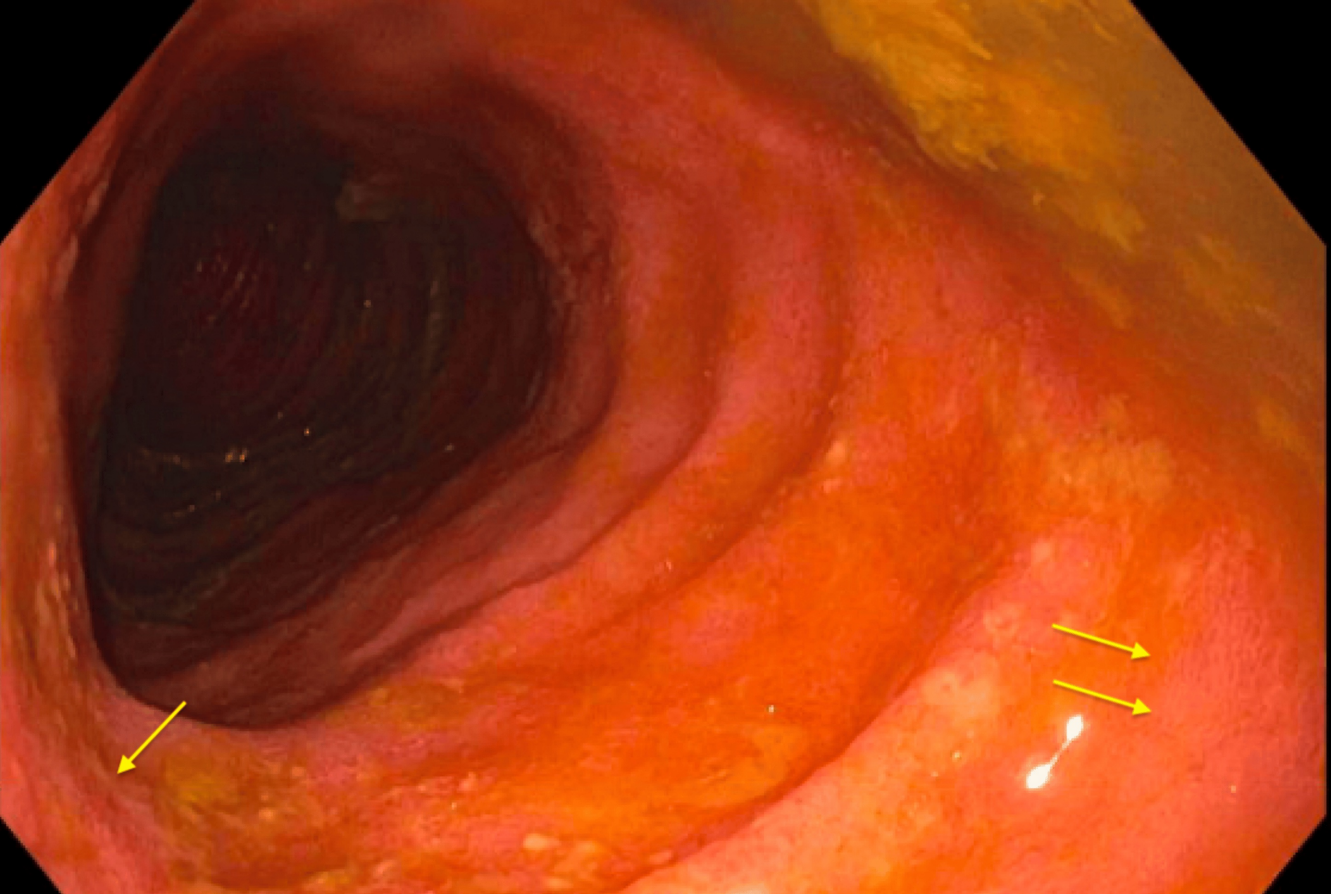
Distal ileum demonstrating granular, congested mucosa with inflammation in distal ileum (yellow arrows).

**Figure 4:**
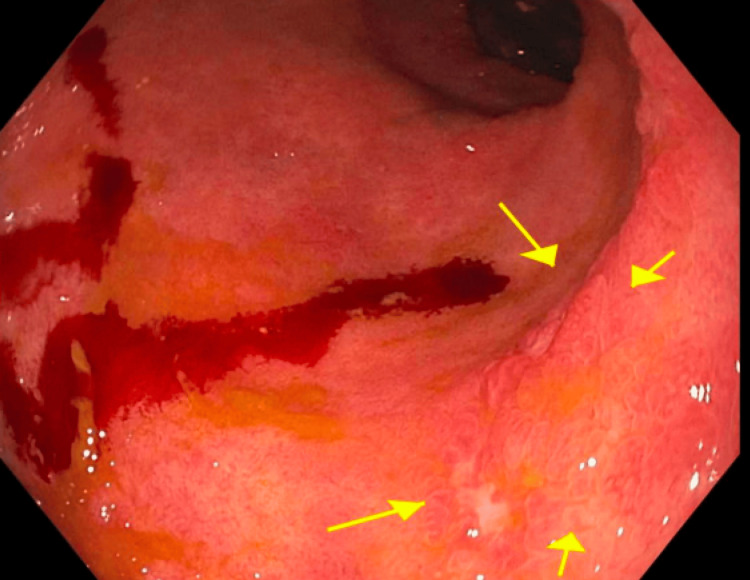
Short segment of sigmoid colon proximal to the anastomosis demonstrating diffusely granular and congested mucosa in sigmoid colon segment. Ulcerations are indicated by yellow arrows. Note: Bleeding is secondary to trauma from biopsy sites.

Upper endoscopy with biopsy was unremarkable with normal gastric and small bowel mucosa. Unprepped colonoscopy done after a tap water enema showed ulceration and inflammatory polyps at the ileosigmoid anastomosis (as shown in Figure 2), scattered ulcers with granular congested mucosa in the distal ileum (as shown in Figure 3), and granular congested mucosa with ulceration in the rectosigmoid colon (as shown in Figure 4).

Biopsies of the rectum showed active Crohn’s disease, and those of the small intestine showed active enteritis. The overall endoscopic appearance was similar to that of the flexible sigmoidoscopy performed six months prior. Inflammation of a specific segment of the colon or rectum alters motility through effects of inflammatory mediators on the enteric nervous system and the muscular layer; patients experience stool buildup and constipation proximal to the site of inflammation. This was also suggested by her feces-filled distal small bowel loops seen on CT scan. Hence, the enteritis on the neo-terminal ileal biopsy was likely due to localized stasis leading to secondary small intestinal bacterial overgrowth (SIBO). Patients with constipation often experience overflow diarrhea as the liquid stool seeps around solid feculent matter. Hence, her baseline higher frequency of bowel movements was thought to be due to altered anatomy, active rectal disease, overflow diarrhea, and SIBO.

**Figure 5:**
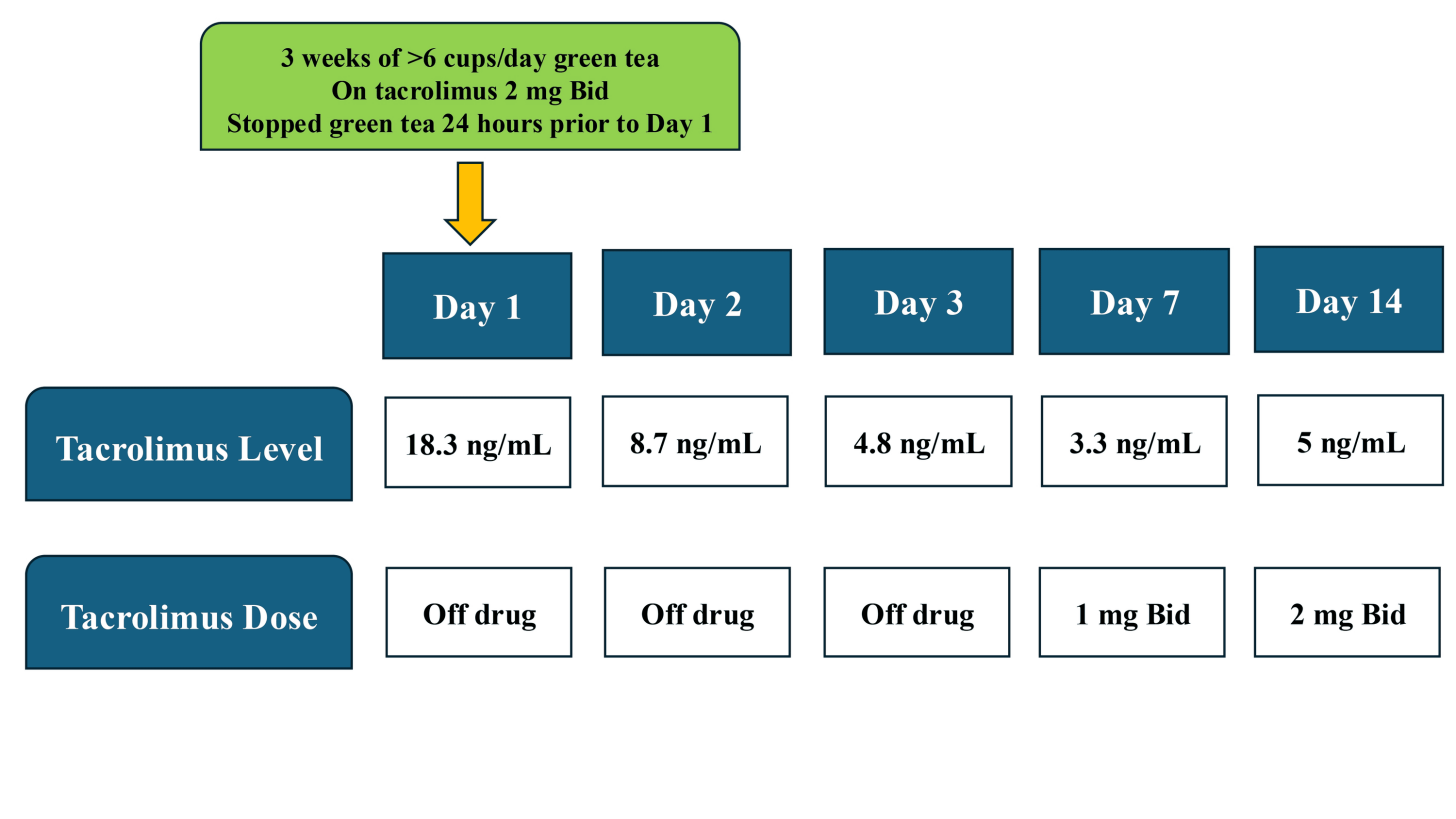
Summary of tacrolimus level and dose trend following discontinuation of green tea. Bid: twice a day; ng: nanogram; mL: milliliter

The patient was started on rifaximin 550 mg twice a day empirically for SIBO. She had stopped consuming green tea two days prior to admission. Her tacrolimus was held due to supratherapeutic levels. Her tacrolimus troughs subsequently dropped to 8.7 ng/mL the next day and 4.8 ng/mL the day after. Her abdominal pain, nausea, and bloating symptoms resolved without any use of steroids within a day of presentation. Her rapid resolution of symptoms coincided with the improvement in supratherapeutic tacrolimus levels. She had only received one dose of rifaximin before symptoms resolved. Tacrolimus IR was restarted at 1 mg twice a day on day 3, and her levels dropped to 3.3 ng/mL on day 7, after which tacrolimus IR was increased to 2 mg twice a day again with levels of 5 ng/mL on day 14. The tacrolimus level and dose trend is summarized in Figure 5.

She maintained therapeutic levels for the next two months on a steady dose after. Given her active disease in the rectum, we planned to discontinue vedolizumab and stop mycophenolate in anticipation of starting guselkumab therapy.

## Discussion

Tacrolimus is a potent immunosuppressant with widespread applications. The oral bioavailability of tacrolimus ranges from 17% to 23% [1]. IR formulations require twice-daily administration and exhibit variable absorption, which is significantly affected by food intake, particularly high-fat meals, thereby reducing peak serum concentrations and overall drug exposure [1]. In contrast, extended-release formulations provide more stable absorption profiles, permit once-daily dosing, and are associated with a prolonged half-life [1]. Gastrointestinal absorption of tacrolimus is further limited by P-gp/ABCB1, an efflux transporter that pumps the drug back into the intestinal lumen, thereby contributing to marked interpatient pharmacokinetic variability [3]. Tacrolimus demonstrates extensive plasma protein binding (~99%) and a large volume of distribution (~30 L/kg) [3]. Metabolism occurs predominantly through hepatic cytochrome P-450 (CYP) enzymes such as CYP3A4 and CYP3A5 isoenzymes, while elimination is primarily biliary, with approximately 93% excreted in feces and less than 1% excreted unchanged in urine [3]. Depending on formulation and patient-specific factors, the elimination half-life ranges from 4 to 41 hours, with an average of approximately 12 hours for IR tacrolimus [3].

Tacrolimus dosing is individualized according to the underlying indication and target trough concentrations. In IBD, trough levels of 10-15 ng/mL during the first two weeks, followed by maintenance levels of 5-10 ng/mL, have demonstrated therapeutic efficacy [4]. Following liver transplantation, target trough concentrations are generally maintained between 7 and 10 ng/mL during the initial postoperative months and are typically reduced to below 5 ng/mL after the first year post-transplant [5]. In living-donor liver transplant recipients, combination immunosuppression with adjunctive agents such as mycophenolate may also be required to prevent rejection [5].

Several factors may increase tacrolimus exposure, including inaccurate laboratory assay measurements, CYP3A5 genetic polymorphisms, concomitant administration of CYP3A inhibitors, renal dysfunction, biliary stasis, and systemic inflammatory states [5,6]. Inflammatory conditions can suppress the activity of drug-metabolizing enzymes and transporters (DMET) through two major pathways: (i) nitric oxide-mediated proteasomal activation resulting in degradation and reduced activity of DMET proteins, and (ii) cytokine-driven transcriptional downregulation during the acute-phase inflammatory response [6,7]. Medications that strongly inhibit CYP3A enzymes and consequently increase tacrolimus concentrations are summarized in Table 2 [1].

**Table 2:** Drugs strongly inhibiting CYP3A enzyme leading to altered tacrolimus pharmacokinetics. CYP: cytochrome P-450; HIV: human immunodeficiency virus; CMV: cytomegalovirus

Drug class	Drugs	Strength of CYP3A interaction
Azole antifungals	Voriconazole, posaconazole, itraconazole, ketoconazole	Strong
Macrolide antibiotics	Clarithromycin, troleandomycin, chloramphenicol	Strong
HIV protease inhibitors	Ritonavir, nelfinavir	Strong
Anti-virals (CMV)	Letermovir	Strong
Calcium channel blockers	Diltiazem, verapamil, nifedipine	Strong
Antidepressant	Nefazodone	Strong

Dietary substances have also been implicated in altered tacrolimus pharmacokinetics. Grapefruit, pomelo, pomegranate, and clementine may increase tacrolimus exposure through inhibition of CYP3A4 and P-gp activity [2]. Herbal supplements, including *Panax ginseng *and *Hypericum perforatum* (St. John’s wort), have likewise been associated with modulation of tacrolimus metabolism via induction or inhibition of CYP isoenzymes and P-gp, although evidence supporting clinically significant interactions remains limited [2]. Green tea contains bioactive catechins and theaflavins that may inhibit CYP enzymes and P-gp transporters; however, existing data are inconsistent, and further investigation is needed to clarify the clinical significance of this interaction [2]. Notably, at least one case report has described elevated tacrolimus levels associated with green tea consumption [8].

Tacrolimus therapy is associated with a broad spectrum of adverse effects involving multiple organ systems. Common toxicities include acute kidney injury, electrolyte disturbances (including hyperkalemia, hypokalemia, hypercalcemia, hypocalcemia, hyponatremia, and metabolic acidosis), blurred vision, arthralgia, alopecia, cutaneous rash, headache, insomnia, hypertension, cardiac arrhythmias, and new-onset diabetes mellitus [1]. Gastrointestinal adverse effects are also frequently encountered and may include abdominal pain, nausea, vomiting, diarrhea, constipation, anorexia, and dyspepsia [1]. Rarely, tacrolimus has been associated with diffuse gastrointestinal ulceration [9]. Histopathologic findings in tacrolimus-associated colitis classically include prominent epithelial apoptosis, neutrophilic cryptitis, and plasma cell-rich inflammation within the lamina propria [10].

In our case, the patient’s bowel frequency, endoscopic findings, and histology had remained stable for months while maintaining therapeutic tacrolimus troughs on a consistent dose. She had no hepatic or renal dysfunction and was not taking any strong CYP3A inhibitors. We therefore suspected that consumption of non-commercial green tea, possibly along with other unidentified additives or extracts, altered tacrolimus metabolism. This was supported by a decline in tacrolimus levels after discontinuing green tea despite continued medication, followed by stable troughs after resuming her prior dose of 2 mg twice daily. Her gastrointestinal symptoms also rapidly resolved with normalization of tacrolimus levels, suggesting supratherapeutic tacrolimus levels as the probable etiology.

However, the inability to perform toxicologic, pharmacokinetic, and phytochemical analyses in a routine clinical setting precludes definitive establishment of causality. In addition, the preparation contained orange extract and potentially other unidentified constituents. The effects of orange-derived compounds on CYP3A-mediated metabolism are not well characterized, and a grapefruit-like interaction cannot be excluded. Accordingly, the observed clinical effects may reflect the combined influence of multiple bioactive components within a non-standardized formulation rather than green tea alone.

## Conclusions

This case highlights a probable interaction between a non-commercial herbal preparation and tacrolimus metabolism, resulting in supratherapeutic drug levels and gastrointestinal toxicity. The patient had stable tacrolimus dosing and therapeutic trough concentrations for several months prior to presentation, without hepatic dysfunction, renal impairment, or exposure to known CYP3A inhibitors. Symptom resolution and normalization of tacrolimus levels following discontinuation of the preparation support a temporal association; however, causality cannot be definitively established. The observed effect may have been related to green tea, orange extract, unidentified additives, or a combination of these components within a non-standardized preparation. Although the specific contributing factor remains uncertain, this case underscores the potential risks of non-commercial herbal products in patients receiving tacrolimus.

Future studies incorporating pharmacogenetic profiling and detailed toxicological or phytochemical analysis of herbal preparations may help clarify potential mechanisms and strengthen causal inference in similar interactions; however, such approaches may not be feasible in routine clinical practice. Increased awareness, careful medication reconciliation, and counseling regarding herbal and dietary supplement use remain essential in this population.
